# Human DNA levels in feces reflect gut inflammation and associate with presence of gut species in IBD patients across the age spectrum

**DOI:** 10.21203/rs.3.rs-6809327/v1

**Published:** 2025-07-07

**Authors:** Chiara Mazzoni, Bracha Ochana, Gili Focht, Esty Harpenas, Ahmad Quteineh, Esther Orlansky Meyer, Ami Ben Ya’acov, Shimrit Shmorak, Ruth Shemer, Eyal Shteyer, Yuval Dor, Moran Yassour

**Affiliations:** Hebrew University of Jerusalem; Hebrew University of Jerusalem; Shaare Zedek Medical Center; Hebrew University of Jerusalem; Shaare Zedek Medical Center; Shaare Zedek Medical Center; Shaare Zedek Medical Center; Hebrew University of Jerusalem; Hebrew University of Jerusalem; Shaare Zedek Medical Center; Hebrew University of Jerusalem; Hebrew University of Jerusalem

**Keywords:** Human DNA, neutrophils, microbiome, IBD, prediction

## Abstract

**Background:**

Feces are a complex matrix that holds precious information regarding gut processes. Comprehensive fecal DNA sequencing is largely utilized as a non-invasive way to profile the gut microbiome, but is majorly overlooked in other fields. Clinical practice and research on Inflammatory Bowel Diseases (IBD) would greatly benefit from accurate and non-invasive methods to monitor gut inflammation in IBD patients. In IBD, immune cell storming and epithelial cell shedding in the gut increase the amount of human DNA in feces, making fecal DNA profiling a desirable approach to monitor gut inflammation dynamics.

**Methods:**

We used a combination of sequencing techniques to comprehensively characterize the fecal DNA diversity in a newly established cohort of IBD patients and Controls (SZ cohort, N=134 children, Israel). We performed methylation-based human cell-specific profiling together with shotgun metagenomics to characterize the human and the microbial DNA content in feces, respectively. Moreover, we included a large external validation cohort (LLDeep+1000IBD cohorts, N=689 adults, the Netherlands) in order to extend our findings from the methylation-based profiling to the more broadly-available quantification of human DNA in metagenomics sequencing.

**Results:**

We found that neutrophil DNA dominates fecal human DNA content in IBD patients, and our measurements were highly correlated with fecal calprotectin levels. Combining neutrophil and other cell type DNA fractions in one metric was able to distinguish between remissive and active cases of IBD. Human reads percentage by metagenomics was well correlated with disease severity and species richness, which had distinct trends in CD and UC over time. We used a combination of species richness, human DNA percentage and microbiome composition data to predict IBD and distinguish CD from UC in both adult and pediatric IBD patient cohorts.

**Conclusions:**

The comprehensive characterization of human and microbiome fecal DNA is a useful approach to track immune response level and investigate the interaction that the immune system has with gut microbiome richness and composition over time, enriching opportunities for better disease monitoring and thus better treatment of IBD patients.

## Introduction

Human feces contain DNA, not only from the trillions of microorganisms inhabiting the gut (microbiome), but also from human cells, which are shed from the epithelium into the lumen as part of their physiological turnover(1–3). In healthy individuals, human DNA content in feces is negligible, usually not exceeding 10% of the total fecal DNA(4,5). However, in case of chronic inflammatory states of the gut, such as in Inflammatory Bowel Diseases (IBD), gut epithelial cells are shed at a faster rate into the lumen(1,6), and several immune cell types migrate to the gut mucosa in response to pro-inflammatory stimuli(7). Several studies already reported that in these and other scenarios, human DNA content in feces increases considerably(8–11). Yet, in metagenomic pipelines for the analysis of the human microbiome, human DNA is commonly considered an unwanted byproduct of shotgun metagenomics. Human DNA reads are removed early on in the analysis, to reduce computational burden, and most importantly, to protect the identity of participants prior to publication in data repositories(4). Many have also proposed techniques for reducing human DNA content in DNA samples, especially where a considerable amount, if not the majority, of the reads are expected to be human, such as in skin, saliva and vaginal swabs (12–14). In this context, most microbiome studies report the number of human reads merely as part of the technical assessment of the sequencing process alone.

Although gut microbiome studies have so far ignored human fecal DNA in their analyses, assessing its quantity and origin could complement and enrich microbiome research, especially studies focusing on IBD. The association between IBD and gut microbiome has been heavily researched since the inception of the microbiome field(15–27), yet finding a nexus between gut microbiome composition and IBD onset and development is still an open question(28–30). Gut microbiome composition changes based on IBD subtype, Crohn’s Disease (CD) and Ulcerative colitis (UC) being the two major ones, and often has been reported to reflect states of disease flare(31). Moreover, gut microbiome variability across IBD patients has been reported to reach over 60%(32), hence searching for the factors that might drive this variability is imperative to further understand and better treat IBD. Indeed, IBD patients are characterized by a large spectrum of inflammatory states, changing over time, differentially managed by several pharmacological regimes(33,34). Analysis of the human DNA found in feces could elucidate, not only on the general inflammatory status of the patients’ gut, but also, on tissue involvement, such as colon and small intestinal injury, as well as immune response level and response to medical treatment. To perform such an in-depth assessment, repeatedly over time on chronically ill IBD patients, human cell profiling needs to be cost-effective and non-invasive. In this regard, quantification of total human DNA is straightforward, since computational alignment of the sequencing reads to a human genome reference is routinely used to discard human reads. Conversely, characterization of the origin of human fecal DNA is more complex, but several approaches are available. The most cost-effective and less invasive ones were initially developed for the analysis of cell-free DNA (cfDNA) in plasma. Indeed, plasma cfDNA sequencing and analysis has been proven to effectively detect tissue involvement in various processes in the body, even remote ones, most often of inflammatory nature(35–39). One of the most recent approaches to the analysis of cfDNA composition is methylation profiling, for which we, and others, have compiled large cell methylation-marker atlases(40,41).

In this study, we performed a combination of human and microbial fecal DNA profiling of IBD pediatric patients and Controls from a newly established cohort, in order to assess a wide variety of IBD-associated features, such as: tissue injury, inflammatory immune response, and luminal microbiome composition. We profiled the microbial community structure together with total human DNA percentage and tissue of origin (using methylation-based profiling). As nearly all publicly-available microbiome cohorts do not include methylation profiles of the human DNA, we explored the generalizability of our findings by looking at the total human reads percentage. For this purpose, we used two external large microbiome cohorts as validation cohorts to examine whether total human reads percentage could serve as a proxy for the more in-depth methylation profiling we performed on our cohort. Multifaceted genomic approaches, such as this one, have the potential to elucidate on novel aspects of host-microbe interaction in the context of chronic gut inflammation, and potentially solve unexplained variability that is observed in the clinical setting.

## Methods

### Patient enrollment and sample collection

Subjects (children, up to the age of 18 years old) were enrolled at the Pediatric Gastroenterology Institute at Shaare Zedek Medical Center, Jerusalem, Israel. Subjects were included in the study according to one of the following criteria: (i) the subject received an IBD diagnosis, (ii) the subject underwent a colonoscopy for blood in feces and diarrhea, but resulted not affected by any other gastrointestinal condition, or (iii) the subject was otherwise healthy (recruited for the purpose of the study at the orthopedic clinic at Shaare Zedek Medical Center). Fecal samples were collected in ethanol by the subjects and kept in a home freezer till the sample could be transferred (within 24 hours) to the hospital, and stored in a −80 C freezer. Samples included in the study amount to 134 in total (Control=29,CD=62,UC=45), with 10 individuals sampled twice, and 9 individuals sampled three times.

IBD unclassified (IBDU, N=11) cases are included in the analysis as UC for three reasons: (i) 10 samples at a later time point were re-classified as UC, (ii) the SZ clinical team assesses IBDU cases with pUCAI disease activity score, (iii) after performing a SZ-specific PCoA we calculated study group centroids and the IBDU centroid was very close to the UC centroid, confirming that at the population level, IBDU cases are indistinguishable from UC patients. Only one IBDU sample (GB_543_1) is included as CD given a later time point is classified as such.

### DNA extraction

Samples were extracted and sequenced in six separate batches (**Supplementary Figure 1b**), where for each batch a *no-sample* control was also produced. We used a custom DNA extraction protocol, optimized to maximize DNA yield, fragment length and microbial lysis, as the same DNA was required for Bisulfite treatment, and shotgun metagenomic sequencing both by Illumina and Oxford Nanopore. Briefly, 200 mg of aliquoted feces were used as input to the DNA extraction protocol, characterized by three separate lysis steps. The three lysis steps include: (i) a 10% SDS-based lysis, (ii) a ProteinaseK-based lysis, and (iii) a bead-beating-based lysis, with optimized power and time settings. In between lysis steps, high-speed centrifugation collects the unlysed pellet at the bottom, leaving the supernatant containing DNA to be collected in a separate tube. At the end of the lysis steps three separate tubes containing DNA in a supernatant are processed separately with a column-based DNA isolation procedure, using the Power Soil Pro Kit (QIAGEN), and eluting DNA in 70 uL of buffer. Performing only one DNA extraction, the protocol yields ~40 mg (median) of total DNA/sample, ensuring enough DNA for sequencing and other DNA-based procedures.

### Methylation profiling of human DNA

Cell-specific and tissue-specific methylation profiling was performed as previously described(5,40). Briefly, cell-specific and tissue-specific biomarkers were identified by searching regions with at least five CpG sites in a minimal window 150 bp long. Identified biomarkers were validated in vitro by testing them with corresponding cell/tissue type genomic DNA samples, and in-silico against a previously published large scale cell methylation atlas(40). DNA samples included in the study were treated with bisulfite using EZ DNA Methylation-Gold^™^ (Zymo Research), according to the manufacturer’s instructions, and eluted in 20 uL. Bisulfite-treated DNA was amplified in a two-step multiplex PCR as previously described(42). Briefly, for each PCR reaction, several sequence primers (30 pairs maximum), including short adapters, were mixed with the input bisulfite-treated DNA to amplify the identified biomarkers. This was followed by an exonuclease step and a second PCR reaction using primers specific to the adapters. This final PCR added sequencing barcodes, hence the PCR products could be pooled together, run on a 3% agarose gel with ethidium bromide staining, and extracted by a Zymo gel recovery kit. Pooled PCR products were sequenced on a MiSeq or NextSeq sequencer for a total depth of 10K reads/sample. Sequenced reads were demultiplexed, and aligned to the biomarker sequences with Bismark, using a computational pipeline available on github (https://github.com/Joshmoss11/btseq). Reads were filtered out when having <80% similarity to a biomarker sequence. Proper bisulfite conversion was assessed checking the expected CpG sites. Bases are considered methylated if “CG” is read, and unmethylated if “TG” is read. Samples were discarded when having less than 1000 total reads. Finally, the fraction of tissue-specific DNA in the sample was calculated as the fraction of molecules in which all CpG sites were unmethylated. Five samples were discarded from the methylation-based analysis because the sum of the different cell type fractions was either <5% or >150%.

### Absolute quantification of human DNA

To quantify the number of human DNA molecules, we used Evergreen ddPCR kit according to manufacturer instructions (BIORAD Droplet Digital^™^ PCR Technology). Primers were designed for the human SFPTC-1 gene. The ddPCR was run on 5ng of fecal DNA and included a negative control (no template control) and a positive control. Manually set cutoff thresholds were used for each sample, according to acceptance criteria defined during the optimization of each reaction (QuantaSoft^™^ software version 1.7.4). We then inferred the percentage of human DNA per ng of DNA, assuming that each copy of human SFPTC-1 identified in stool represented one genome equivalent, or 3.3 picograms of human DNA. These fractions were used to normalize cell-type specific fractions to the total human DNA percentage.

The primers for the ddPCR reaction were:

SFTPC1: 5’- AGC AAA GAG GTC CTG ATG GAG A-3’ (forward), 5’- GCA GGG CCC ATC ACA CAC AT-3’ (reverse).

### Shotgun metagenomic sequencing, preprocessing and taxonomic classification

Shotgun metagenomic sequencing libraries were prepared using Nextera DNA Library Prep Kit by employing half of the reagents’ volume and total input DNA. For each sequencing run a *no-template* control was also included. Libraries were sequenced at a target depth of 8M reads/sample with 150 bp single-end reads on a Miseq or Nextseq machine.

Reads were quality filtered using fastq-mcf (-q 10 -l 75 --qual-mean 20; https://github.com/ExpressionAnalysis/ea-utils), human reads were identified by using bowtie2(43) and samtools(44) (-f 4) against the human genome assembly (GRCh38.p13), quantified and removed. Non-human reads were taxonomically classified using Metaphlan 4(45) (database v. mpa_vJan21_CHOCOPhlAnSGB_202103). All analyses on microbiome composition were performed at the species-level.

### Clinical information processing

#### SZ cohort

Clinical information for all subjects was obtained from RedCap. Pediatric-specific disease activity scores, such as pUCAI and pCDAI, were used to assess the severity of symptoms. These are numerical scores that are categorized in levels by using standard conversion tables into *remission, mild, moderate* and *severe*. For the purpose of this paper, all categories other than *remission* were combined into the new category *active*.

#### 1000IBD cohort

We were provided with some basic metadata about the subjects, including disease activity scores such as Harvey Bradshaw index (for CD) and the Simple Clinical Colitis Activity index (for UC). For harmonization purposes, these indices were first converted from numerical to categorical following standard tables, and then divided into *remission* and *active* as indicated above.

#### All cohorts

All samples had the total number of reads and the number of human reads quantified to calculate the percentage of human reads by metagenomics, used throughout. Fecal calprotectin values were capped at 2100 mgc/g whenever the value was above this threshold.

### Statistical analyses

The analyses (all performed in R) aimed at characterizing the differences between Control and IBD subtypes and the associative relationship between microbiome composition and human DNA percentage. Principal Coordinate Analysis (PCoA) was performed using robust Aitchison distance on unfiltered relative abundance data to account for compositionality, using *vegdist*() and *prcomp*() functions. Species alpha diversity was assessed using the Shannon index as implemented in *diversity(index = “shannon”)* within the *vegan* package. Correlation analyses were throughout performed using Spearman’s rank correlation coefficients, testing the monotonic relationships between variables, using *stat_cor*() within the *ggpubr* package.

Statistical inference was performed throughout using non-parametric Mann-Whitney rank-sum tests, using *stat_compare_means*() within the ggpubr package or *wilcox_test*() by rstatix package. Specifically, in classifying the 188 species in one of six categories (IBD/CD/UC-lost, IBD/CD/UC-expanded; Figure 3a), in order to determine the direction of statistical difference between groups, we separately tested two different sets of hypotheses, with the Control group as reference group: (i) *loss* and (ii) *expansion. Loss* was tested by setting the parameter *alternative = “less”*, which corresponds to testing the following alternative hypothesis: Median of Group 1 (CD or UC)<Median of Control. *Expansion* was tested by setting the parameter *alternative = “greater”*, which corresponds to testing the following alternative hypothesis: Median of Group 1 (CD or UC)>Median of Control. Finally, p values were corrected for multiple hypotheses testing using the Benjamini–Hochberg procedure. When more than one category could be applied to a single species, the category with lowest Benjamini–Hochberg-corrected p value was reported.

### Linear-mixed models

We modelled fecal calprotectin by running *lmer*() function by the R package *lmerTest* as following: *FecalCalprotectin ~ Human.reads.percentage + disease_activity_level + treatment_advancement*_*only for SZ*_ *+ Age + (1|subject)*_*only for SZ*_. Samples having fecal calprotectin reaching 2100 mgc/g were excluded from the model, given these were artificially capped at this value. Treatment advancement, as included in the model, was conceived as a numerical variable apt to capture the clinical treatment tier the patient had been treated with at the time of sampling, assigning numbers to the treatments categories, ordered by well known stratified treatment regimes: 0 = “None”, 1 = “ASA (oral and rectal), antibiotics or dietary supplement, 2 = “steroids”, 3 = “immunomodulators”, 4 = “immunosuppressants or biologics”.

After having identified neutrophils as the main source of human DNA, we remodelled fecal calprotectin only for the SZ cohort as following: *FecalCalprotectin ~ neutrophils_percentage_normalized_by_Human + disease_activity_level + treatment_advancement + (1|subject) + (1|Age)*.

### XGboost models

All XGBoost models built using cross-validation, for both relative abundance and presence-absence data (CV, Figure 4a) were run using *xgb.cv*() function by *xgboost* R package, on the the LLDeep+1000IBD cohort using the following parameters (nfold = 5, stratified = TRUE, nrounds = 200, objective=‘binary:logistic’, eval_metric = ‘auc’, eta = 0.05, gamma = 1, lambda = 3, nthread = 3, max_depth = 10, min_child_weight = 1, subsample = 0.8, colsample_bytree = 0.8, prediction = T). ROC curves were plotted using *ggroc*() function by *pROC* R package.

Feature importance according to *Gain, Cover* and *Frequency* for all models was extracted by, first rerunning each model at its best round with xgb.train() function and then using xgb.importance() function. Additionally we performed SHAP analysis by using *SHAPforxgboost* R package. First we used *shap.values*() function to retrieve mean SHAP values, and then *shap.prep*() function for retrieving sample-specific values.

Validation of the CV models was run on the SZ cohort using *predict*() function and SHAP analysis was run as previously. Age and Sex were excluded from the training variables in all models, given that the CD group in the SZ cohort is both younger and lower in male subjects.

## Results

### Fecal human DNA quantification by different methods correlates with standard metrics of inflammation

**Figure 1:**
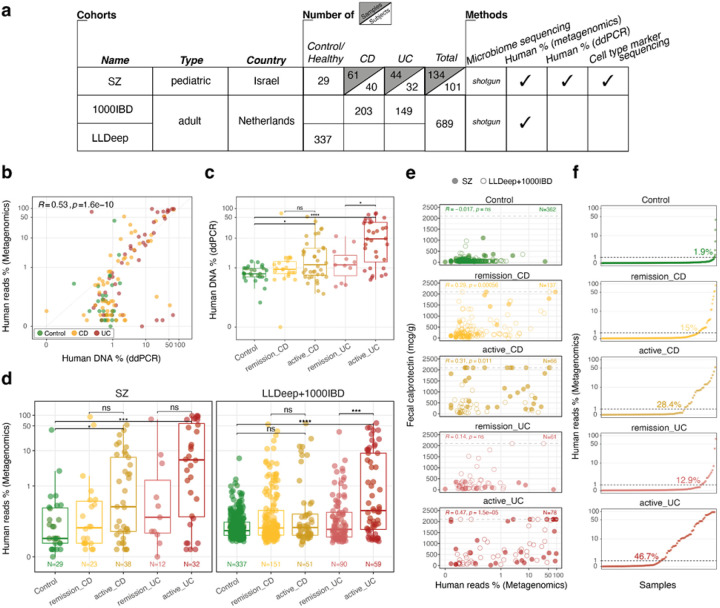
Human DNA content in feces from IBD patients is a proxy for disease activity level **a**. Overview of cohorts, number of samples & subjects, and methods included in the study. **b**. Human DNA percentage as calculated by two methods and their correlation (Spearman): percentage of human DNA as evaluated by ddPCR (Methods; x-axis), percentage of metagenomic reads mapping to the human genome (y-axis; GRCh38.p13 assembly). c. Human DNA percentage (ddPCR) across study groups. **d**.Human reads percentage (Metagenomics) across study groups by cohort. **e**. Correlation (Spearman) between human reads percentage (Metagenomics) and fecal calprotectin. f. Fraction of samples having >1% human reads percentage (Metagenomics) across disease activity levels, with increased values in active cases of IBD. All comparisons are statistically significant at level 0.05 after Mann-Whitney rank-sum test: p<0.05: ‘*’, p<0.01: ‘**’, p<0.001: ‘***’, p<1e-04: ‘****’.

To explore the relationship between gut microbiome and human DNA level in feces, we performed comprehensive microbial and human DNA profiling for a pediatric IBD cohort established at Shaare Zedek Medical Center in Jerusalem (SZ cohort)(5). For this study, we sequenced 134 fecal samples from 101 children (median age=15), including Crohn’s Disease patients (CD; 40 children), Ulcerative Colitis patients (UC; 32 children), and Control subjects not affected by gut inflammatory conditions (27 children; Figure 1a). Samples were collected together with detailed clinical information and pediatric-specific disease scores evaluating disease activity (pCDAI and pUCAI; see Methods). We combined shotgun metagenomic sequencing with two independent methods for human DNA-specific quantification and profiling (Figure 1a): (i) a multiplex digital droplet PCR (ddPCR) to quantify the total human DNA content in feces(39), and (ii) a methylation-based marker approach for the quantification of several different human cell types and tissues in feces(40,46) (Methods). In addition, the results from the ddPCR assay were used as a normalization factor for the methylation-based method.

In order to validate and generalize our findings to larger, already published microbiome cohorts, we included the analysis of two other published cohorts of Dutch adults, the 1000IBD cohort(47) (IBD=351) and the LLDeep-followup cohort(48) (Control=337; Figure 1a). Specifically, we wanted to check whether our human DNA analysis in feces could be recapitulated by using human reads percentage by shotgun metagenomics, which comes at no additional cost and is available in most microbiome-specific sequencing studies.

We started with comparing the different human DNA quantification methods to evaluate whether they performed consistently across fecal samples, and found that human reads percentage by metagenomic sequencing was largely well correlated to the human DNA percentage measured by ddPCR (Figure 1b). Then, using pCDAI and pUCAI scores (see Methods), we checked if disease activity had an impact on the amount of human DNA in feces, and found that, on average, human DNA was higher in active cases of both CD and UC compared to Controls and remission IBD, with quite large variability among patients, especially in active UC cases (Figure 1b–c). The fraction of patients with human DNA over 1% was the highest in active cases of both CD and UC (28% for CD and 47% in UC), modest in remission cases (15% in CD, 13% in UC) and negligible in Controls (2%; Figure 1e).

As human DNA amount in feces correlated with disease activity, we hypothesized human DNA could also be correlated with fecal calprotectin, a standard biomarker of intestinal inflammation(49). While the two measures correlated better in active IBD cases compared to remissive cases (Figure 1d), we proceeded to test whether human reads percentage by metagenomics could predict fecal calprotectin values across the different study groups. For this, we employed linear-mixed models, one for each cohort location independently (SZ-Israel and 1000IBD+LLDeep- the Netherlands), including disease activity and treatment advancement (when available), while adjusting for age and subject identity (for the longitudinal SZ cohort). In our models, human reads percentage and disease activity alone explained a fair amount of the variation in fecal calprotectin values (SZ R2=0.33, LLDeep+1000IBD R2=0.29), but when treatment advancement was included in the model for the SZ cohort (see Methods), the proportion of variance explained by the model increased significantly (R2=0.53). In the model, treatment advancement was negatively associated with fecal calprotectin, as indicated by its negative coefficient estimate (β = −159.04, p << 0.05, **Supplementary Table1**; Methods), highlighting how much clinical treatment based on biological therapy or immunosuppressants influence immune response, specifically fecal calprotectin levels. Overall, human DNA percentage in feces could be a useful additional parameter to assess inflammatory status, although factors such as clinical treatment can determine large variance observed within and across the study groups.

### Fecal DNA origin informs on IBD inflammation levels

**Figure 2:**
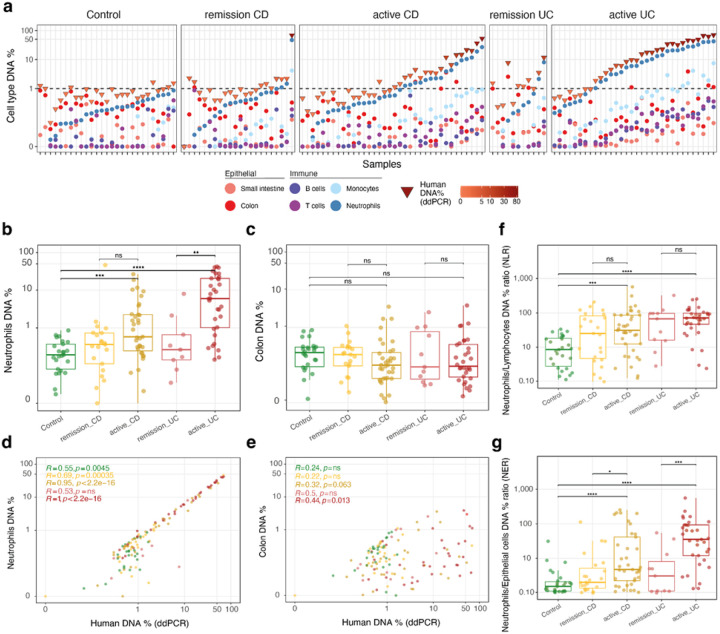
Cell type profiling of feces from IBD patients reveals the majority of human DNA comes from neutrophils **a**. Methylation-based cell- and tissue-specific DNA percentage across samples in different disease activity groups. **b**. Neutrophil DNA and **c**. Colon DNA percentage across study groups, only neutrophil DNA can distinguish Control from active IBD d. Neutrophil DNA and **e**. Colon DNA percentage correlation (Spearman) with total human DNA percentage (ddPCR). **f**. Neutrophil/Lymphocyte (B and T cells) DNA percentage ratio (NLR) from feces. **g**. Neutrophil/Epithelial cells (small intestine and colon) DNA percentage ratio (NER) as an alternative to NLR. All comparisons are statistically significant at level 0.05 after Mann-Whitney rank-sum test: p<0.05: ‘*’, p<0.01: ‘**’, p<0.001: ‘***’, p<1e-04: ‘****’.

As we found more fecal human DNA in active cases of IBD, we imagined that investigating the origin of human DNA in feces would elucidate how different tissues and cell populations are involved in the gut inflammatory process. By using methylation-based markers previously developed(5,39), we assessed DNA content from different tissues, including colon and small intestine, and several different cell populations, such as leukocytes (neutrophils and monocytes), and lymphocytes (B and T cells), resulting in an estimation of these cell percentages across all samples (Figure 2a, Methods). Among the cell populations profiled, neutrophils were the most abundant in feces of IBD patients, and they could differentiate not only Controls from IBD, but also remissive from active cases of UC (Figure 2a–b, **Supplementary Figure 2a**). Neutrophil levels correlated almost perfectly with the total human DNA amount, especially where both measures were above 1%, reaching up to 50% of the total human DNA in some cases (Spearman R=0.88, p << 0.001 for all samples together; Figure 2c). Neutrophil DNA percentage also correlated well with human reads percentage as inferred by metagenomic sequencing (overall Spearman R=0.64, p << 0.001; **Supplementary Figure 2b**).

As calprotectin is the most abundant protein in neutrophils and gets released into the lumen after cell burst(50,51), we next wondered whether our measured neutrophil percentage could reliably predict fecal calprotectin levels, better than just human DNA percentage, as examined in the previous section. We evaluated this with a linear-mixed model only for the SZ cohort, for which neutrophil DNA quantification was available (see Methods, **Supplementary Table 1**), and added similar clinical variables as before. Notably, this model clearly outperformed the previous model and explained the majority of the variance within the data (R2=0.68). Conversely, when we flipped the model, and used fecal calprotectin measurements to predict neutrophil levels, our model explained almost all the variance within the data (R2=0.90). While neutrophil DNA was highest in active IBD, colon DNA was either the first or second most abundant component in Controls and remissive IBD, but it could not differentiate Controls from IBD patients, since its values mainly ranged between 0–10% of total human DNA across all patients (Figure 2c, **Supplementary Figure 2c**).

To check whether we could detect complex dynamics involving several cell compartments in the gut, we tried to combine the different tissues and cell types measurements into one single metric. For this purpose, we first calculated Neutrophil–Lymphocyte ratio (NLR), as it has been previously suggested to be differentiating endoscopic activity in IBD patients when measured by cell counts from peripheral blood(52–54) (Figure 2f). Next, we calculated Neutrophil-Epithelial ratio (NER), exploiting the unique capability of our approach to quantify epithelial cells (Figure 2g). For both CD and UC, NER outperformed NLR in its ability to differentiate remissive from active IBD cases.

In summary, methylation-based profiling exposed neutrophil DNA dominance in the human DNA fraction in feces, and comprehensively allowed us to characterize several cell fractions in an integrative way. Evaluating the levels of different cell populations in the gut, as they are released in the lumen, allowed us to distinguish the different study groups, along the inflammatory spectrum.

### Species count drives sample variance in IBD and across age ranges

**Figure 3:**
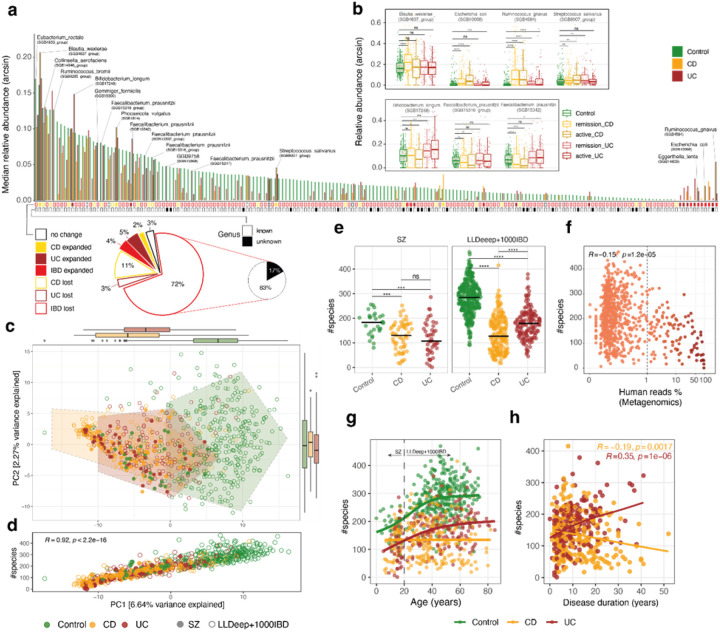
Gut microbiome richness drives variance across IBD and Control subjects and differentially correlates with disease duration in CD and UC **a**. (top) Median relative abundance (arcsine-transformed) of species with median relative abundance >0% in at least one study group (N=188). Several species of interest are named and highlighted; (**bottom**)Breakdown of the 188 species in six different categories, based on their expansion or loss in CD, UC or IBD in general (Methods). **b**. Relative abundance across study groups for species previously known to be increased in IBD patients (**top**)and species here reported to be increased in IBD (**bottom**; Mann-Whitney rank-sum test). **c**. Principal Coordinate Analysis (PCoA) on species-level gut microbiome composition data (CLR-transformed relative abundance) as profiled by Metaphlan (v.4.1; Methods). **d**. Species number correlation (Spearman) with the first principal component (PC1) of the PCoA. e. Species number by study group across SZ and LLDeep+1000IBD cohorts (Mann-Whitney rank-sum test). **f**. Species number correlation (Spearman) with human reads percentage (Metagenomics). **g**. Species number trends across ages and h. over disease duration. All comparisons are statistically significant at level 0.05 after Mann-Whitney rank-sum test: p<0.05: ‘*’, p<0.01: ‘**’, p<0.001: ‘***’, p<1e-04: ‘****’.

With respect to gut microbiome composition, it has been well established that reduced microbial diversity is a hallmark of gut inflammation(55–57). Here, we wanted to assess whether microbial diversity in IBD was correlated with human DNA levels as measured and described in the previous sections, and whether we could find similar trends in both pediatric and adult IBD patients. To characterize in detail the differences in microbial profiles between IBD patients and Controls, we first looked at the overall distribution of median relative abundance across species, ordered by Control values (N=188, Figure 3a, **top** arcsine-transformed, **Supplementary Table 2**). We could divide the species into six groups, based on whether they were expanded or lost in both or either one of the IBD groups (Figure 3a, **bottom**). For example, *B. longum* and *F. prausnitzii* SGB15342 were preferentially expanded in UC, while *B. wexlerae* and *S. salivarius* were uniquely expanded in CD (Figure 3b). Notably, the single species that had the highest median relative abundance and the highest relative drop in both CD and UC was *R. bromii* followed by *G. formicilis* (6th & 18th highest in Control, respectively). Amongst the species with the lowest median relative abundance in Controls, we found species such as *R. gnavus*, *E. coli* and *E. lenta* expanded in both IBD groups (Figure 3a, **right**). Species lost in either UC or CD accounted for 14% of the total (26 out of 188), while species lost in both CD and UC accounted for 72% of the species pool (135 out of 188). Notably, 17% of the species lost in both CD and UC were of unknown genera, to date solely identified computationally and described by metagenomic-derived gene markers(45) (GGB-SGBs). In particular, the highest ranking among them was GGB9758_SGB15368, located at the 37th place from the top of the distribution (Figure 3a, **middle**).

To compare pediatric and adult IBD patients according to their gut microbiome composition, we then performed a Principal Coordinate Analysis (PCoA, Aitchison distance on relative abundance data), to visualize sample clustering and dispersion. Visually, pediatric patients (SZ cohort), adult patients (1000IBD cohort) and Control subjects formed a continuum, where most IBD patients clustered furthest away from Control subjects (Figure 3c). Permutational analysis of variance (Permanova) of the gut microbiome composition confirmed that IBD patients were statistically different from Controls (adonis p < 0.001), but also revealed that pediatric patients and adult patients could be distinguished from each other (adonis p = 0.001). This was not surprising, since the gut microbiome in childhood is still in development, and early IBD onset could disturb the natural process of microbiome maturation(58,59). To understand how much of the observed variation could be explained by microbial richness, we calculated the correlation between the first principal component in the PCoA (PC1) and species count, calculated as number of species with median relative abundance >0% (Figure 3d, **Supplementary Figure 3a**; also done with Shannon index, shown in **Supplementary Figure 3b-c**). Notably, species count was better correlated with PC1 than Shannon index (Spearman R=0.92 and R=0.84, respectively), indicating that presence and absence of species might drive the majority of the variability across Control and IBD gut microbiome samples. Indeed, when examining species count distributions, we found that overall the species count in Controls was roughly double than in IBD patients (overall: median_Control_=274.5, median_CD_=128.5, median_UC_=168.0; Figure 3e, **Supplementary Figure 3d**), but the difference was more pronounced in adults than in children. Moreover, in pediatric IBD patients, species count differences between CD and UC were not statistically significant, possibly driven by the fact that children overall have a less mature microbiome. In parallel to the drop in species count, IBD patients were also characterized by increased human DNA amount (above 1%) when species count was below 200 (Figure 3f, **Supplementary Figure 4a**). Species count was also negatively correlated to fecal calprotectin levels and NER, as calculated in the previous section (**Supplementary Figure 4b-c**). Lastly, species count was found to be positively correlated with the total number of non-human reads in the samples (**Supplementary Figure 4e**). To investigate whether the reduction in species count observed in IBD patients could be solely explained by differences in the number of non-human reads, we subsampled all samples to 1 Million non-human reads and recalculated the gut microbiome profile and species count (**Supplementary Figure 4f**). The results confirmed that the number of non-human reads did not artificially inflate the species count difference between IBD patients and Controls, highlighting that the high content of human DNA and reduced species richness are both biological features of the IBD-associated gut contents.

Finally, we looked at the role of age and disease duration in determining species count levels. It is well established that children have a maturing gut microbiome(60), but surprisingly we found a positive trend between species count and age way beyond adolescence, reaching stabilization only around 40 years old (Figure 3g). This trend was shared between Controls and UC patients, where the latter stayed at a lower species count over time. CD patients lacked any positive trend between species count and age, suggesting that the gut microbiome in these cases is more severely impaired than in UC. When looking at species count since IBD diagnosis, species count in UC had a positive correlation with disease duration, while CD had a negative correlation, highlighting there could be a cumulative negative effect of inflammation on species richness over time (Figure 3h).

Overall, we assessed the spectrum of gut microbiome species changes between IBD patients and Controls, highlighted known and unknown species that are either expanded or lost in IBD and the power of species count in recapitulating Control-to-IBD gut microbiome sample variance across age ranges and disease duration.

### Presence and absence of species predicts and characterizes IBD subtypes

**Figure 4:**
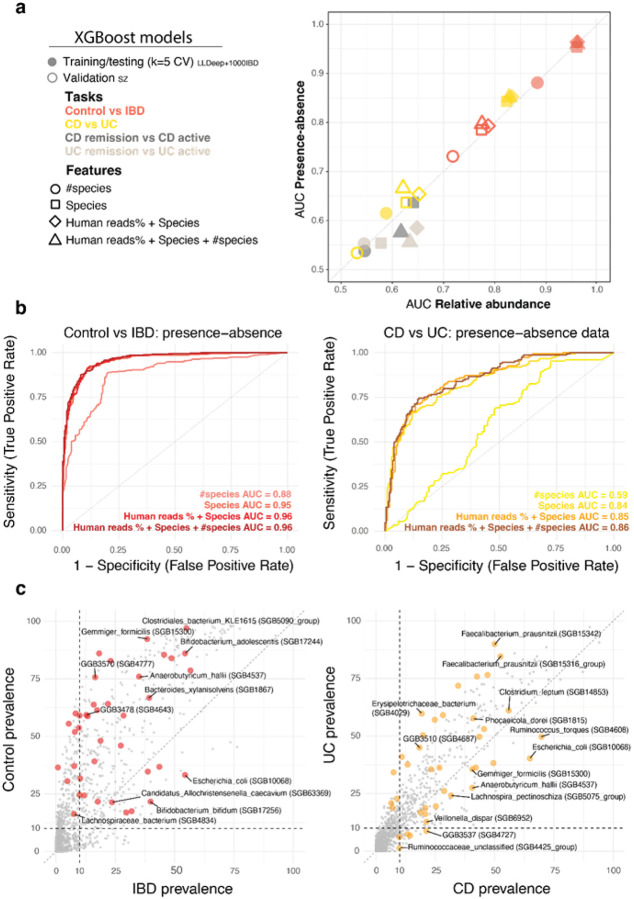
XGBoost models trained on presence-absence species data and human reads percentage accurately predict adult IBD and moderately translate to pediatric patients **a**. Performance of XGBoost tree-based machine learning models (AUC values) when using relative-abundance data (x-axis) versus presence-absence data (y-axis). Each point represents a model trained on a specific combination of Features (shapes) and for different Tasks (colors). Performance comes from either 5-fold Cross-Validation (full, on LLDeep+1000IBD) or external validation (empty, on SZ cohort) **b**. ROC curves of presence-absence data models, color coded by feature combination, in predicting Control vs IBD (**left**) and CD vs UC (**right**). **c**. Species prevalence across IBD subjects (x-axis) vs. Control subjects (y-axis) (**left**) and CD subjects (x-axis) vs.UC subjects (y-axis) (**right**)in LLDeep+1000IBD cohort exclusively. The top twenty species with the highest importance across prediction models are colored.

We proceeded to examine whether we could use the microbiome data together with the human reads percentage to predict sample phenotypes. Specifically, we built tree-based machine learning models using Extreme Gradient Boosting (XGBoost) to either predict IBD, classify the IBD subtypes (CD or UC), or within these subtypes, distinguish between remissive versus active states of the disease. As input, we used species count (*#species*) together with percentage of human reads (*Human reads* %) and species composition data (*Species*; presence-absence or relative abundance; Figure 4). We trained and tested the XGBoost models in 5-fold cross validation (CV) on the LLDeep+1000IBD cohort, to exploit the sample size advantage. Then we validated the models’ performance on the SZ cohort, despite knowing their performance would be suboptimal, given the overall differences between adult and pediatric subjects mentioned in the previous section. Yet, we deemed informative investigating how much the adult microbiome-based models would generalize to the pediatric microbiome samples. To our surprise, XGBoost models trained on either relative abundance or presence-absence data of the species had practically the same performance (Figure 4a), with few exceptions for models in the RemissivevsActive task, which performed better using relative abundance data, but overall underperforming with AUCs < 0.66. Across CV and independent validation, species count alone had lower AUC values compared to the other more complex models including full species data, although it exceeded our expectations in the ControlvsIBD task (AUC = 0.88; Figure 4ab). The models including more than species count alone had very similar performances to each other (AUC ⩾ 0.95; Figure 4b), with none to little contribution by human reads percentage in the ControlvsIBD and CDvsUC tasks, respectively. However, human reads percentage was often among the most important features according to performance metrics such as Gain, Frequency, or SHAP values (**Supplementary Figure 5a-b**). As expected, performance in the independent validation on pediatric microbiome samples was lower than in the CV, with 0.16 AUC difference for the most complete model (*Human reads % + Species + #species*; **Supplementary Figure 5c**). Since the performance of models using only presence-absence profiles was as good as the full microbial composition, we decided from here on to only use the presence-absence information.

To identify the features that were most discriminative in each task, we performed SHAP analysis across the different models (**Supplementary Figure 5X**), and found that the top 20 features had at least 10% prevalence within the IBD or the Control groups (LLDeep+1000IBD cohort, Figure 4c). To mention a few, *B. bifidum* and *E. coli* were more prevalent in IBD cases than in Controls, while *F. prausnitzii* species (SGB15342 and SGB15316) were more prevalent in UC compared to CD (Figure 4c). Using these predictive models for IBD, we showed that species count alone is quite informative and correlates with the level of inflammation as assessed by human DNA content in feces. We appreciated how species presence-absence data was as predictive as relative abundance data, highlighting how, in IBD microbiome profiling, as well as other disease contexts, accurate detection of species can be more important that precise estimation of relative abundance.

## Discussion

Fecal contents are considered a reflection of the physiological processes taking place along the gastrointestinal tract, on both the host and the gut microbiome side. On the host side, analyses of feces provide information on food consumption, absorption and metabolism(61–63), as well as immune system status and response to transient or chronic infection(64). On the microbiome side, microorganisms secrete metabolites that can influence a plethora of host-related processes, even beyond the gut, hence studying their composition and dynamics provides an important key to interpret their role in human physiology(65–74). In this study, we combined analysis on both the host and gut microbiome side, in order to investigate their interaction in the context of chronic Inflammatory Bowel Diseases (IBD).

On the host side, we applied methylation-based profiling of human fecal DNA in IBD patients and Controls to explore the value of identifying human DNA tissue-of-origin in the characterization of the immune status of individuals. Genomics-based approaches, such as methylation-based human DNA profiling, can represent an alternative to traditional immunodetection methods for the characterization of cell-level and tissue-level processes in the body. For example, fecal calprotectin is released from dying neutrophil cells(50,51), and it is commonly quantified as a non-specific biomarker of gut inflammation (75–77). Here, we used the methylation-based approach to directly quantify neutrophil cells death, which yielded effective and sensitive measurements, especially in cases of high-inflammation background, such as severe IBD patients, where the standard fecal calprotectin quantification method is limited by its detection range.

Our methylation-based profiles indeed confirmed neutrophil storming to the gut mucosa and lumen to be a dominant process during inflammation(78,79), which aligns with fecal calprotectin values, as we showed with linear regression analysis. Moreover, we highlighted that neutrophil DNA prevails over colon DNA, despite the fact that feces are formed along the colon and rectum and cell epithelial shedding is a known response to inflammation(80,81). Nevertheless, we cannot exclude that other processes, such as DNA degradation in the gut environment during inflammation, might specifically reduce epithelial DNA concentration in feces. In the absence of any element for physical protection, epithelial cells’ DNA could be degraded(82), or even consumed by microorganisms scavenging for nutrients(83,84). However, neutrophil DNA can be particularly well shielded from degradation, given that it is extruded from neutrophils together with other proteins, creating antimicrobial web-like structures called Neutrophil Extracellular Traps (NETs)(85,86). Indeed, the inflamed gut is the stage of sophisticated immune warfare, which includes *nutritional immunity*, where host immune cells deplete the environment of certain molecules that are essential for microorganisms growth and survival (e.g. iron, zinc and manganese) (87). In response, microorganisms have evolved known sophisticated strategies to circumvent starvation(88–90), and perhaps additional mechanisms specifically target the reuse of host cells’ DNA. Moreover, our methylation-based profiling could be expanded to more cell types of the gut epithelium, opening avenues for a more rapid and cost-effective alternative to single-cell approaches such as cell sorting(91–95). Methylation-based biomarkers could be developed, not only for less abundant cells, such as goblet and paneth cells, but also for specific functional immune subtypes that uniquely expand in case of inflammation, such as aged neutrophils(96), inflammatory macrophages, myeloid-derived immunosuppressive cells or exhausted T cells(97–102).

On the microbiome side, we investigated how species richness relates to disease severity, human fecal DNA level and disease course over time, highlighting species that expand or drop in IBD. Specifically, we mentioned *B. longum and B. bifidum*, as expanded and more prevalent in UC patients, respectively. Both *B. longum and B. bifidum* are among the most well established pioneer species of the healthy infant gut microbiome(103–106), and as such we do not expect them to be implicated in the pathogenesis of IBD. Additionally, we mentioned *R. gnavus* as expanded in both UC and CD. *R. gnavus* is a known IBD-associated species(107,108), but also a known infant gut colonizer, with recently reported infant-specific clades (109,110). In all these cases, higher abundance and prevalence in IBD do not necessarily associate with strictly pathogenic traits. The presence of these species might however be explained by their great capacity of metabolizing glycans, HMOs in the infant gut(111–114), and mucin-derived glycans in the inflamed gut (115–117). Indeed, in the context of gut inflammation, where survival conditions are at the hardest and species are in a race for survival, species presence correlates strongly with their metabolic capacity to endure challenging conditions (118,119), and although some studies have already suggested this, there is the need to expand research in this direction.

Finally, in predicting IBD, we showed presence-absence of species to be as predictive as relative abundance data, in line with other reports(120). This should encourage the use of presence-absence data in microbiome analysis over relative abundance data, especially in cases where relative abundance is less reliable. For instance, relative abundance can be misleading in disease contexts where microbial load drastically changes across samples, due to frequent bowel movements causing DNA content dilution(121). For the same reason, several studies have suggested the use of absolute abundance over relative abundance(122–125). Moreover, differential analysis of relative abundance has been reported to yield very different results based on which method was used to perform it, hence clearer consensus on the most appropriate methods to use would be also needed to minimize reporting spurious results(126).

Ultimately, this study approaches the important aspect of non-invasive, microbiome-based diagnostics(127), which other studies have also recently addressed, with various predictive learning approaches (128–130).

To date, most microbiome studies on IBD have investigated pediatric and adult IBD cohorts separately, without much emphasis on characterizing the IBD-associated microbiome as it relates to age(32,131,132). Although we understand the value of keeping the age of the target study group as narrow as possible, here we decided to incorporate a large set of external microbiome samples to cover the entire age spectrum and to be able to compare the pediatric to the adult IBD patients. This age-inclusive approach gave us the opportunity to identify overarching characteristics of IBD, such as changes in species richness across ages, which have been largely overlooked by other microbiome studies, but are an important topic of discussion in the clinical setting(133). Our comparative analysis was accompanied by a range of challenges and limitations, such as: (i) small number of Control pediatric subjects, (ii) heterogeneity of the patients with respect to clinical and geographical variables(134), (iii) differential availability of metadata, including medications, detailed disease location, IBD family history, and disease activity scores, (iv) disregard to viral gut microbiome diversity(135–139). Nevertheless, we deemed it important to present analyses that would address some important aspects related to IBD development over time, such as the interaction between species richness and disease duration. In our view, these kinds of analyses can help identify how the gut microbiome responds and contributes to gut inflammation and how it can be treated or modified to ameliorate symptoms and reduce life-long complications(140,141).

## Conclusion

This study integrates comprehensive microbiome profiling with detailed human DNA quantification and cell-type characterization, providing a dual perspective on host-microbiome interactions in the context of Inflammatory Bowel Diseases (IBD). We employ a methylation-based approach to settle previous speculations regarding the origin of human DNA in feces during gut inflammation and we combine it with shotgun metagenomic sequencing to identify overarching gut microbiome characteristics across pediatric and adult IBD patients. We showed that human DNA content in feces primarily comes from neutrophils and correlates with IBD activity level and fecal calprotectin measurements. We described the expansion and loss of IBD-associated bacterial species but at the same time highlighted how presence-absence microbiome data alone can predict IBD as well as relative abundance data. We believe the findings of this study could have applications in the non-invasive monitoring of patients’ inflammatory status, and that our methylation-based profiling could be further tailored to experimentally investigate the expansion of more recently discovered cellular subpopulations during inflammation.

## Supplementary Material

Supplementary Files

This is a list of supplementary files associated with this preprint. Click to download.

- SupplementaryTables.xlsx
- SupplementaryFigures.docx

## Data Availability

The human-filtered metagenomic sequencing data for the SZ cohort generated in this study will be deposited in the SRA database under BioProject PRJNA1265906. Metadata pertaining to the SZ cohort is provided in **Supplementary Table 3**. The use of LLDeep and 1000IBD cohorts in this study respects the form agreed with Lifelines and the UMCG Department of Genetics. Metagenomic sequencing data and basic metadata for the LLDeep cohort was provided under a Data Access Agreement with the UMCG Department of Genetics and access to the data was granted through the following EGA Dataset Accession Number: EGAD00001006959. Metagenomic sequencing data and clinical metadata for the 1000IBD cohort was provided under a Data Transfer Agreement with the University Medical Center Groningen and Prof. Dr. R.K. Weersma, and access was granted to metadata at EGAD00001003991 and metagenomic sequencing data at EGAD00001004194.

## Abbreviations

IBD: Inflammatory Bowel Disease
CD: Crohn’s Disease
UC: Ulcerative Colitis
SZ: Shaare Zedek Medical Center
NLR: Neutrophil/Lymphocyte ratio
NER: Neutrophil/Epithelial ratio
XGBoost: eXtreme Gradient Boosting
NET: Neutrophil Extracellular Trap
